# Prevalence and progression of pneumonia in immunocompetent adults with varicella

**DOI:** 10.1186/s12985-024-02303-3

**Published:** 2024-02-09

**Authors:** Ran Cheng, Fei Lin, Zhonghua Deng, Jingjin Liang, Xiaoguang Li, Ming Lu, Lu Li

**Affiliations:** 1 Department of Infectious Diseases, Peking University Third Hospital, Beijing, China; 2 Department of Respiratory and Critical Care Medicine, Peking University Third Hospital, Beijing, China

**Keywords:** Varicella, Immunocompetent, Adult, High-resolution computed tomography, Pneumonia

## Abstract

Pneumonia is the most common complication of varicella infections. Although previous studies have tended to focus mainly on immunocompromised patients, varicella pneumonia can also occur in healthy adults. Therefore, in this study, we aimed to assess the progression of varicella pneumonia in immunocompetent hosts. This retrospective study involved immunocompetent adult outpatients with varicella who attended the adult Fever Emergency facility of Peking University Third Hospital from April 1, 2020, to October 31, 2022. Varicella pneumonia was defined as a classic chickenpox-type rash in patients with infiltrates on chest computed tomography. The study included 186 patients, 57 of whom had a contact history of chickenpox exposure. Antiviral pneumonia therapy was administered to 175 patients by treating physicians. Computed tomography identified pneumonia in 132 patients, although no deaths from respiratory failure occurred. Seventy of the discharged patients were subsequently contacted, all of whom reported being well. Follow-up information, including computed tomography findings, was available for 37 patients with pneumonia, among whom 24 reported complete resolution whereas the remaining 13 developed persistent calcifications. Notably, we established that the true incidence of varicella pneumonia is higher than that previously reported, although the prognosis for immunocompetent hosts is generally good.

**Supplementary Information:**

The online version contains supplementary material available at 10.1186/s12985-024-02303-3.

## Introduction

Varicella, a highly contagious disease often experienced in childhood, is associated with fever and a characteristic vesicular rash that occurs mainly on the trunk, head, and face [1]. The resulting skin vesicles are filled with infectious virus particles that eventually aerosolize, thereby transmitting the varicella-zoster virus to healthy individuals via the nose and mouth [1]. Adults are more prone to severe infections than are healthy children [2–4]. Varicella vaccination provides effective protection and significantly reduces the associated disease burden [5–8]. Since the introduction of treatment with two doses of varicella attenuated live vaccine (VarV), the incidence of varicella has declined [9–11], and to date, VarV has been adopted in routine vaccination programs for children in 36 countries and regions, including the United States, Australia, and Germany [5, 12]. However, given concerns regarding cost-effectiveness, this varicella vaccine has not been implemented in China, and notably, during the period from 2005 to 2019, the annual average incidence of varicella in China increased by more than 60% [13, 14]. This trend is a cause for concern, as although varicella is generally a mild and self-limiting disease, it can give rise to severe complications.

By definition, pneumonia is an infection of the lungs or pulmonary parenchyma [15], which is the most frequent serious complication of varicella in adults [16, 17], in whom pneumonia has been reported to occur in < 5 to 50% of all cases of varicella infection [18–22]. Whereas most studies on adult varicella pneumonia have tended to focus on immunocompromised patients and pregnant women [16, 23–25], this complication can also occur in immunocompetent adults [26]. Accordingly, in this study, we sought to establish the rates of consecutive varicella pneumonia in immunocompetent adult patients and document the resulting disease prognoses.

## Materials and methods

### Patients

Peking University Third Hospital is a large teaching hospital with an Infectious Diseases Unit. The Unit contains an emergency facility for fever patients, referred to as the Fever Emergency facility. During the past 3 years, chest computed tomography (CT) scans have been performed for most patients initially admitted to this facility for the purposes of COVID-19 screening. For patients with varicella, chest CT scans were typically performed on days 1 to 5 after development of a rash.

In this retrospective study, we enrolled 193 immunocompetent outpatients with varicella who had visited the adult Fever Emergency facility of Peking University Third Hospital from April 1, 2020, to October 31, 2022. This facility is staffed by infectious medicine and internal medicine residents, supervised by attending physicians, and for the purpose of this study, we reviewed the facility’s logbooks to identify patients ≥ 18 years of age diagnosed with chickenpox or varicella. These logbooks contained information pertaining to patient demographic data, clinical features, contact history, risk factors for pneumonia, degree of hypoxia, need for assisted ventilation, disposition, and development of secondary complications. A particular sign or symptom was assumed to be absent if not documented in the medical records. The decision to administer antiviral therapy was made by the treating physicians (senior residents and attending physicians).

The diagnosis of varicella is based on the appearance of a vesicular pruritic skin rash that occurs mainly on the trunk, head, and face. Patients were classified as having pneumonia if they had a positive chest CT scan, which was defined by the presence of small nodules with surrounding ground-glass attenuation, patchy ground-glass attenuation, or a coalescence of lesions. Positive chest images were interpreted and finally verified by a radiologist.

Patient medical records were reviewed for the documentation of follow-up visits. If no chest images were available, telephone follow-ups were conducted with a view toward persuading these patients to consider being re-examined with a chest CT scan and thereby ensure that they had fully recovered.

### Statistical analysis

Statistical analysis was conducted using SPSS for Windows (version 24.0, IBM, Armonk, NY). A two-tailed *P* < 0.05 was considered significant. Continuous variables with a normal distribution were reported as the mean ± standard deviation, and non-normal variables were presented as medians and interquartile ranges (IQRs). Categorical variables were expressed as frequencies (percentages). Differences between two cohorts were compared using the independent samples *t*-tests for continuous variables and the *χ*^2^ tests for categorical variables.

## Results

### Patient characteristics

Among the 193 immunocompetent patients diagnosed with varicella vesicular pruritic skin according to the outpatient data of Peking University Third Hospital, 186 patients met the study’s age and clinical disease criteria and were accordingly included for further analyses (Additional file 1: Fig. S1). None of the infected patients received immunosuppressive therapy or cytotoxic agents for the treatment of varicella. All records assessed in this study are available for review.

The baseline characteristics of the study population are summarized in Table 1. Of the 186 patients, 137 (74.7%) were male and 46 were current smokers. None of the enrolled women were pregnant, although one was lactating. 143 (76.9%) of the patients were uncertain as to whether they had been vaccinated against chickenpox. The mean patient age was 24.27 ± 6.76 years (range, 18–46 years). The mean time between the appearance of a rash and fever was 1.65 days. Contact history revealed that 57 (30.6%) patients had been exposed to chickenpox. For 175 patients, the treating physicians provided antiviral chemotherapy, including acyclovir, valaciclovir, and famciclovir. All patients underwent a chest CT scan, and pneumonia was identified in 132 (70.9%) of them. One patient was hospitalized on account of suspected pancreatic injury due to varicella. None of the enrolled patients were diagnosed with varicella encephalitis, and none died as a consequence of infection.

**Table 1:** Baseline characteristics of patients with varicella

Parameters	Total (*n* = 186)
Age (year)	24.27 ± 6.76
Male, *n* (%)	137 (74.7%)
Contact history, *n* (%)	57 (30.6%)
Antiviral chemotherapy, *n* (%)	175 (94.1%)
Pneumonia, *n* (%)	132 (70.9%)
Mean duration between rash and fever (days)	1.65 ± 1.09
WBC (× 10^12^/L)	4.27 (1.82)
NE (%)	63.7 (12.67)
LY (%)	23.85 (8.83)
Admission rate (%)	1(0.5%)
Mortality rate (%)	0

*WBC* white blood cell, *NE* Neutrophil, *LY* Lymphocyte

### Patients with pneumonia

**Fig. 1:**
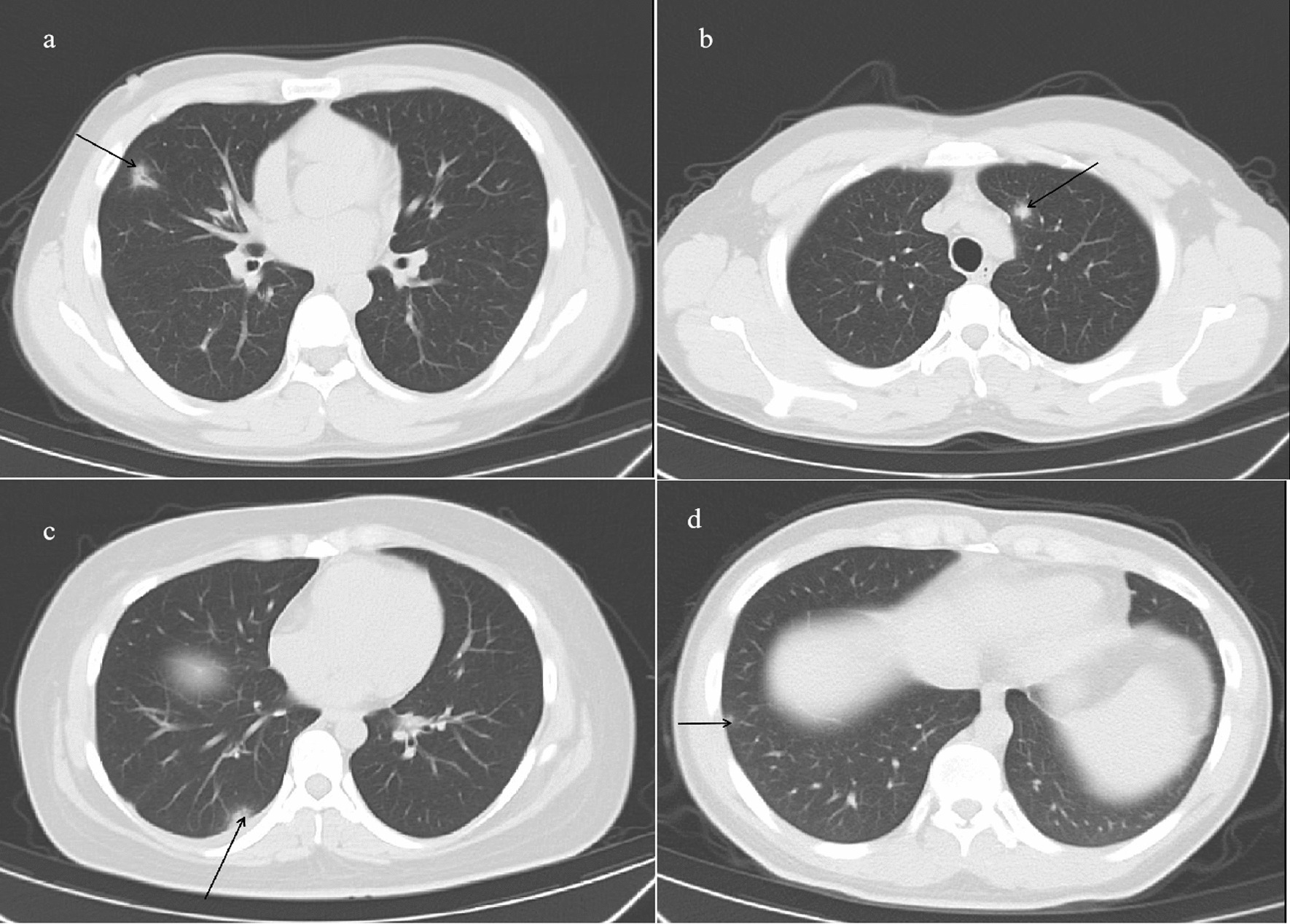
A high-resolution computed tomography scan showing an irregularly shaped nodular opacity with surrounding ground-glass attenuation **a** and **b** A subpleural nodule and small nodule were detected in the lower right lobe **c** and right lobe **d**, respectively

The mean age of patients with pneumonia was similar to that of patients without pneumonia (24.08 ± 6.96 versus 24.61 ± 6.43 years, respectively, *P* = 0.420). No significant differences were detected between the patients with and without pneumonia with respect to age, presence of a cough, headache, chest pain, fatigue, or history of lung disease (Table 2). Other specific signs and symptoms of potential varicella-associated complications were documented only infrequently. None of the patients with pneumonia died of respiratory failure. Current smokers (38, 20.4%) were found to have an increased risk of developing varicella-zoster pneumonia, whereas seven (3.8%) showed evidence of chronic lung disease or previous lung damage (chronic obstructive pulmonary disease, asthma, previous pneumonia, or tuberculosis) and risk factors for pneumonia. Figure 1 presents details of the selected cases of varicella pneumonia.

**Table 2:** Difference between varicella patients with and without pneumonia

Parameters	patients with pneumonia (*n* = 132)	patients without pneumonia (*n* = 54)	*P*-value
Age	24.13 ± 6.92	24.63 ± 6.41	0.420
Male	104 (78.8%)	35 (64.8%)	0.047*
Current smoking	38 (28.8%)	8 (14.8%)	0.045*
Cough	24 (18.2%)	14 (25.9%)	0.234
Headache	56 (42.4%)	22 (40.7%)	0.833
Fatigue	27 (20.5%)	13 (24.1%)	0.586
Chest pain	5 (3.8%)	1 (1.9%)	0.498
Lung disease	7 (5.3%)	1 (1.9%)	0.292

*P < 0. 05

Seventy (51.9%) of the 132 patients with pneumonia who were discharged from the Fever Emergency facility were subsequently contacted, all of whom reported being well following the varicella infection. Follow-up information, including CT scans, was available for 37 of the 132 patients with pneumonia. During a mean follow-up period of 156.7 days, 24 of these patients reported a complete resolution of pneumonia. Among the remaining 13 patients, the lesions calcified and persisted indefinitely as randomly scattered dense calcifications in otherwise normal lungs.

## Discussion

Compared with children, adults with varicella infections tend to have a higher frequency of complications, particularly respiratory and neurological conditions, of which pneumonia is the most commonly reported [27]. Given that radiography and CT scans are generally considered unnecessary in the event of unremarkable findings based on physical examinations of the lungs, the true incidence of varicella pneumonia is difficult to assess. In this study, the enrolled patients tended to be young without any underlying chronic disease. They were medically evaluated and could be included in retrospective case series analyses. Among the 186 patients for whom records were assessed, the reported incidence of pneumonia was 70.9%, and all underwent chest CT scans, which could readily detect asymptomatic pneumonia. Respiratory symptoms (such as cough and chest pain) were not considered indicators of pneumonia in this study.

Varicella pneumonia is characterized by a wide range of clinical manifestations, ranging from asymptomatic through mild illness to death, with a mortality rate of between 11 and 20% [28–30]. Histological features of pneumonia associated with chickenpox include an interstitial mononuclear inflammatory infiltrate associated with intra-alveolar proteinaceous exudate, hyaline membrane formation, and type II cell hyperplasia (diffuse alveolar damage) [29]. Chest radiography has revealed that varicella pneumonia causes the development of multiple 5 to 10-mm ill-defined nodules that may be confluent and appear and disappear from different areas of the lungs [28, 29, 31, 32]. Comparatively, high-resolution CT facilitates a more precise detection of varicella-zoster pneumonia. Previously reported high-resolution CT findings of varicella pneumonia in immunocompetent individuals have revealed a range of manifestations, including small nodules, nodules with surrounding ground-glass attenuation, patchy ground-glass attenuation, and consolidation [33]. Pleural effusions may also occur during the acute phases of the disease but are unusual and were observed in only one patient assessed in the present study. Previous studies have reported that small consolidations generally resolve within a month after the disappearance of the rash. In a few patients, however, the lesions tend to persist indefinitely as randomly scattered, 2- to 3-mm calcifications in otherwise normal lungs [28, 29, 31, 32]. In the present study, among the 37 patients assessed over a mean follow-up period of 156.7 days, 24 reported the complete resolution of pneumonia, whereas the remaining 13 patients were characterized by persistent calcifications. Antiviral treatment might, nevertheless, contribute to the regression of persistent nodules.

Healthy adults and immunocompromised patients who develop varicella should receive treatment. The antiviral agents licensed for varicella treatment include acyclovir, valaciclovir, and famciclovir; the latter two are preferred owing to their higher bioavailability [34, 35]. Treatment is generally administered daily for 7–10 days and should be tailored based on individual clinical assessments [36]. If feasible, antiviral therapy is recommended within 24 h of the initial appearance cutaneous lesions. However, it should still be initiated if patients are later observed to be presenting with the continual appearance of new lesions. In the present study, 175 (94.1%) of the enrolled patients received antiviral treatment. Seventy patients who reported pneumonia were contacted after discharge; all of them reported being well, and the small, round consolidations in these patients resolved within 3 months.

Early antiviral treatment could improve prognosis and reduce mortality for immunocompetent adult patients. According to previous studies, pregnant women with varicella pneumonia have been established to be at a greater risk of a severe clinical course and death [37, 38]. Although acyclovir is not licensed for use during pregnancy, a prospective follow-up of 1,207 women receiving acyclovir failed to detect any significant increase in birth defects compared with those expected in the general population [39]. Regardless, early treatment of varicella at any stage of pregnancy is considered essential and potentially lifesaving.

Despite these important findings, the current study does have certain limitations. First, this was a single-center retrospective study. Second, we only examined a subgroup of patients with skin manifestations, whereas varicella can occur in the absence of a skin rash. Third, we may have missed patients with varicella who had been given other primary diagnoses. Fourth, we were unable to obtain follow-up data for approximately half of the patients, which may have biased the study findings.

## Conclusions

Based on the findings of this study, it appears that the true incidence of varicella pneumonia is higher than that reported previously; however, in the case of immunocompetent hosts, the prognosis is generally good. Adults without significant hypoxia or respiratory distress secondary to pneumonia and without significant comorbid disease or other varicella-associated complications can probably be treated safely as outpatients with oral acyclovir, valaciclovir, or famciclovir administration and subsequent close follow-up.

### Supplementary Information

**Additional file 1.** Supplementary Figure.

## Data Availability

All data are incorporated into the article.

## References

[1] Kennedy PG, Gershon AA (2018). Clinical features of varicella-zoster virus infection. Viruses.

[2] Gershon AA, Breuer J, Cohen JI (2015). Varicella zoster virus infection. Nat Rev Dis Primers.

[3] Gershon AA, Chen J, Gershon MD (2015). Use of saliva to identify varicella zoster virus infection of the gut. Clin Infect Dis.

[4] Nagel MA, Gilden D (2014). Update on varicella zoster virus vasculopathy. Curr Infect Dis Rep.

[5] Marin M, Marti M, Kambhampati A, Jeram SM, Seward JF (2016). Global varicella vaccine effectiveness: a meta-analysis. Pediatrics.

[6] Hirose M, Gilio AE, Ferronato AE, Ragazzi SL (2016). The impact of varicella vaccination on varicella-related hospitalization rates: global data review. Revista paulista de pediatria orgao oficial da Sociedade de Pediatria de Sao Paulo.

[7] Seward JF, Watson BM, Peterson CL (2002). Varicella disease after introduction of varicella vaccine in the United States, 1995–2000. JAMA.

[8] Marin M, Meissner HC, Seward JF (2008). Varicella prevention in the United States: a review of successes and challenges. Pediatrics.

[9] Shapiro ED, Vazquez M, Esposito D (2011). Effectiveness of 2 doses of varicella vaccine in children. J Infect Dis.

[10] Lopez AS, Zhang J, Marin M (2016). Epidemiology of varicella during the 2-dose varicella vaccination program—United States, 2005–2014. MMWR Morb Mortal Wkly Rep.

[11] Bialek SR, Perella D, Zhang J (2013). Impact of a routine two-dose varicella vaccination program on varicella epidemiology. Pediatrics.

[12] Varela FH, Pinto LA, Scotta MC (2019). Global impact of varicella vaccination programs. Hum Vaccin Immunother.

[13] Sui HT, Li JC, Wang M, Liu YM, Yin DP (2019). Varicella epidemiology in China, 2005–2015. Chin J Vaccines Immun.

[14] Dong Pumei WM, Yanmin L (2020). Epidemiological characteristics of varicella in China, 2016–2019. Chin J Vaccines Immun.

[15] Lanks CW, Musani AI, Hsia DW (2019). Community-acquired pneumonia and hospital-acquired pneumonia. Med Clin North Am.

[16] Popara M, Pendle S, Sacks L, Smego RA, Mer M (2002). Varicella pneumonia in patients with HIV/AIDS. Int J Infect Dis.

[17] Ong EL, Mulvenna P, Webb KA (1991). Varicella-zoster infection in adults with cystic fibrosis: role of acyclovir. Scand J Infect Dis.

[18] Wilkins EG, Leen CL, McKendrick MW, Carrington D (1998). Management of chickenpox in the adult. A review prepared for the UK advisory group on chickenpox on behalf of the British Society for the study of infection. J Infect.

[19] Nilsson A, Ortqvist A (1996). Severe varicella pneumonia in adults in Stockholm County 1980–1989. Scand J Infect Dis.

[20] Mohsen AH, Peck RJ, Mason Z, Mattock L, McKendrick MW (2001). Lung function tests and risk factors for pneumonia in adults with chickenpox. Thorax.

[21] Clark GP, Dobson PM, Thickett A, Turner NM (1991). Chickenpox pneumonia, its complications and management. A report of three cases, including the use of extracorporeal membrane oxygenation. Anaesthesia.

[22] Hockberger RS, Rothstein RJ (1986). Varicella pneumonia in adults: a spectrum of disease. Ann Emerg Med.

[23] Fehr T, Bossart W, Wahl C, Binswanger U (2002). Disseminated varicella infection in adult renal allograft recipients: four cases and a review of the literature. Transplantation.

[24] El-Daher N, Magnussen R, Betts RF (1998). Varicella pneumonitis: clinical presentation and experience with acyclovir treatment in immunocompetent adults. Int J Infect Dis.

[25] Smego RA, Asperilla MO (1991). Use of acyclovir for varicella pneumonia during pregnancy. Obstet Gynecol.

[26] Weber DM, Pellecchia JA (1965). Varicella pneumonia: study of prevalence in adult men. JAMA.

[27] Ruben FL, Nguyen ML (1991). Viral pneumonitis. Clin Chest Med.

[28] Triebwasser JH, Harris RE, Bryant RE, Rhoades ER (1967). Varicella pneumonia in adults. Report of seven cases and a review of literature. Medicine.

[29] Fraser RG, Pare J, Pare PD, Fraser RS, Genereux GP (1990). Diagnosis of diseases of the chest. Lung India.

[30] Picken G, Booth AJ, Williams MV (1994). Case report: the pulmonary lesions of chickenpox pneumonia–revisited. Br J Radiol.

[31] Feldman S (1994). Varicella-zoster virus pneumonitis. Chest.

[32] Shirai T, Sano K, Matsuyama S (1996). Varicella pneumonia in a healthy adult presenting with severe respiratory failure. Intern Med.

[33] Kim JS, Ryu CW, Lee SI, Sung DW, Park CK (1999). High-resolution CT findings of varicella-zoster pneumonia. AJR Am J Roentgenol.

[34] McDonald EM, de Kock J, Ram FS (2012). Antivirals for management of herpes zoster including ophthalmicus: a systematic review of high-quality randomized controlled trials. Antivir Ther.

[35] Tyring SK, Beutner KR, Tucker BA, Anderson WC, Crooks RJ (2000). Antiviral therapy for herpes zoster: randomized, controlled clinical trial of valacyclovir and famciclovir therapy in immunocompetent patients 50 years and older. Arch Fam Med.

[36] Lin WR, Lin HH, Lee SS (2001). Comparative study of the efficacy and safety of valaciclovir versus acyclovir in the treatment of herpes zoster. J Microbiol Immunol Infect.

[37] Zambrano MA, Martínez A, Mínguez JA, Vázquez F, Palencia R (1995). Varicella pneumonia complicating pregnancy. Acta Obstet Gynecol Scand.

[38] Esmonde TF, Herdman G, Anderson G (1989). Chickenpox pneumonia: an association with pregnancy. Thorax.

[39] Feldman S (1994). Varicella-zoster virus pneumonitis. Chest.

