# Supplementary figures and images for: Prevalence and progression of pneumonia in immunocompetent adults with varicella
Source: Virol J. 2024 Feb 9;21:39. doi: 10.1186/s12985-024-02303-3 (PMC10858607; doi:10.1186/s12985-024-02303-3)

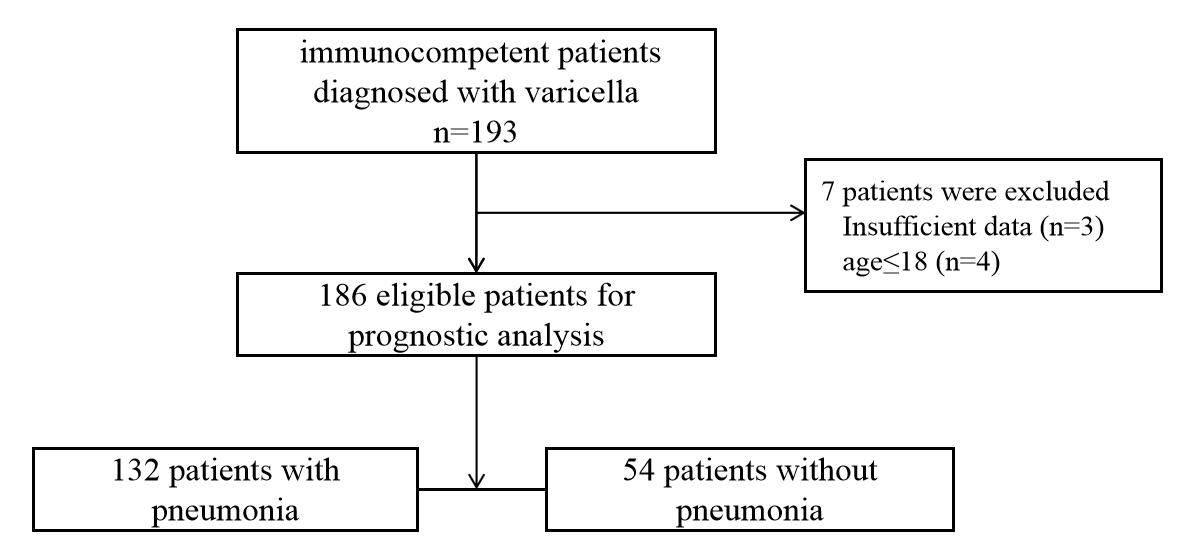

Supplement: Supplementary file 1 — Additional file 1. Supplementary Figure. [file 12985_2024_2303_MOESM1_ESM.tif]
